# Optical Mapping Of Cardiac Arrhythmias

**Published:** 2003-10-01

**Authors:** Rishi Arora, Mithilesh K Das, Douglas P Zipes, Jianyi Wu

**Affiliations:** Krannert Inst. of Cardiology, Indiana University School of Medicine, Indianapolis, IN, USA

## Recent Advances In Mapping Of Cardiac Arrhythmias

The concept of mapping rhythmic activation of the heart dates back to the beginning of last century, with initial descriptions of reentry in turtle hearts [1], to the first systematic mapping of sinus rhythm and then atrial flutter by Lewis et al [2]. Barker et al [3] were the first to map the human heart. Initial mapping was primarily performed using single probes to record activation in different regions of the heart. The 1960’s and 70’s saw the development of computerized mapping of the human heart, e.g. in the cure of Wolf-Parkinson-White syndrome as well as in the study of Langendorff preparations [4]. In fact, most of the recent advances in cardiac mapping have focused on improvements in multisite recordings within the heart, with the ability to simultaneous record electrical activation from several hundreds of sites having contributed significantly to our understanding of atrial and ventricular arrhythmias.

Despite these recent advances, multisite contact mapping suffers from several limitations, including the technical problems associated with amplification, gains, sampling rates, signal-to-noise ratio, and the inability to see signals during high-voltage shocks. In addition, an intrinsic limitation of current mapping techniques is their inability to provide information about repolarization characteristics of electrically active cells, thereby limiting our ability to study entire action potentials. In fact, intracellular microelectrode recordings are still considered the gold standard for the study of action potential characteristics in whole tissue. Microelectrode techniques are limited however, by an inability to record action potentials from several sites simultaneously, thereby precluding their use in high-density activation mapping.

In part due to the above-mentioned limitations, the last few years have seen the development and use of voltage-sensitive dyes as a means to map not only activation, but repolarization as well. Voltage-sensitive dyes, when excited, provide an optical signal that mimics an action potential and thus allows the visualization of both activation and recovery processes in any region under view. This allows one to precisely evaluate the propagation of a wave of excitation and to measure its wavelength visually.

Optical mapping techniques use imaging devices such as a photodiode array or a charge-coupled device video camera with the heart being illuminated and either continuously or spatially scanned. The basis for these techniques is the use of voltage-sensitive dyes that bind to or interact with cell membranes.

## Voltage-Sensitive Dyes

Voltage-sensitive dyes are molecules that bind to the cell membrane with high affinity. While bound to the cardiac cell membrane, the dye molecules fluoresce light in direct proportion to transmembrane voltage. Therefore, voltage-sensitive dyes function as highly localized transducers of membrane potential, transforming a change of membrane potential into a change in fluorescent intensity [5]. For any given constant excitation light intensity and wavelength, light is emitted by voltage-sensitive dyes over a range of wavelengths that can be represented by emission spectra; the emission spectra for a voltage-sensitive dye molecule changes with membrane potential. A suitable filter then passes light only above a certain wavelength, with the amount of light changing at different membrane potentials, thereby allowing the generation of an optical action potential. Importantly, the precise shape of the emission spectra (and therefore the optical action potential) does not correspond to any absolute voltage, and only relative potential change is detected. Also, an optical action potential represents a multicellular spatial average of transmembrane potential from cells within a volume of tissue [6]. Thus, as magnification decreases (1 x), a decrease in the rate of rise of the optical action potential upstroke may occur due to spatial averaging. However, at higher magnifications, the upstroke of the optical action potential approaches that measured from a single cell [7].

The styryl dye, Di-4-ANNEPS, is now the probe of choice for a number of laboratories that are studying complex patterns of activity in the heart. This dye works by the principle of electrochromism, whereby when provoked by excitation light, the molecule undergoes a charge shift from the ground state to the excited state; fluorescence occurs due the emission of a photon during transition from the excited to the ground state. The wavelength of the emitted photon is determined by the change in molecular energy when passing from the excited state to the ground state, less the energy lost in the process. Di-4-ANNEPS possesses several advantages, compared to most other optically sensitive dyes; it exhibits large fractional fluorescence changes during an action potential (8-15%) with low toxicity and photobleaching, and its signal amplitude and kinetics are stable for 2 to 4 hours without restaining the preparation [8].

## Optics And Photodectors

As mentioned earlier, voltage-sensitive dye must be excited by light to induce fluorescence. The most common excitation light sources are tungsten-halogen lamps, mercury arc lamps and argon ion lasers. The source of each optical action potential is derived from as few as one to as many as hundreds of cardiac cells, depending on the extent of optical magnification used. Therefore, a single optical action potential represents the average potential from a small aggregate of neighboring cells.

**Figure 1:**
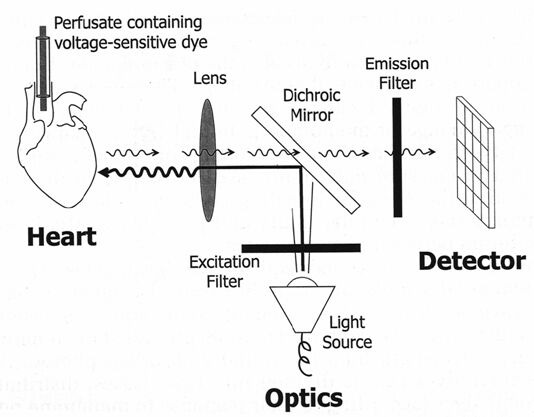
Typical optical mapping system consisting of three major components: 1) the heart preparation; 2) a system of optics; and 3) a detector. An excitation filter is used to pass selected wavelenths of light from the light source to a dichroic mirror, which semiselectively reflects light of this wavelength and directs it toward the preparation. In response to excitation light, voltage-sensitive dye molecules bound to the heart cells fluoresce light in proportion to the membrane potential of the cell to which they are bound. Light emitted from the dye has a longer wavelength, and therefore passes through it (it is not reflected) the dichroic mirror, undergoes a final stage of filtering, and is focused onto the detector. Reprinted with permission from Rosenbaum D. Optical Mapping of Cardiac Excitation and Arrhythmias: A Primer. In Rosenbaum D, Jalife J, ed. Optical Mapping of Cardiac Excitation and Arrhythmias. Oxford: Blackwell Futura Publishing; 2001:2-7

High-quality images can be acquired with a microscope or with photographic lenses. Fluorescence microscopes are commercially available with objective lens magnifications ranging from 4x to 100x, thereby allowing mapping of very small preparations (1 to 5 mm). Photographic lenses are better suited for magnifications under 10x, permitting one to map action potentials from larger preparations (5 to 50 mm). A simple photographic lens system is shown schematically in Figure 1. For very small preparations, laser-scanning systems can be used with a single photodetector, without the need for a collector lens.

The most widely used photodetectors in optical mapping are photodiode arrays and charged coupled device (CCD) video cameras. Both types of photodetectors are similar in that they transduce light energy into electricity. When photons of sufficient energy strike a detector material, electron-hole pairs are created (i.e. the photoelectric effect). Photodiode arrays typically consist of several hundred individual photodiodes, and are configured to instantaneously convert photoexcited charge carriers to current flow (i.e. photocurrent). The magnitude of photocurrent is directly proportional to the light intensity falling on a single photodiode element, and is converted to a voltage signal using a current-to-voltage amplifier; this voltage signal has a amplitude that is proportionate to membrane potential. A major advantage of a photodiode array is that it generates photocurrent in response to membrane potential changes continuously, allowing one to digitally sample the action potential at very rapid rates (i.e. high sampling rates), without compromising the fidelity of the recorded action potential. As a result, these detectors are very useful for measuring details in the time course and morphology of the action potential. They are limited by the number of photodetectors on the array (typically 256 or less).

Unlike a photodiode array, a CCD camera can contain hundreds of thousands of pixels, permitting greater spatial resolution between recording sites. CCD detectors differ from photodiodes in that photoexcited charge carriers are collected within a single pixel over a finite period of time (integration time), and are read off at regular time intervals (i.e. the frame rate). Given that a CCD array contains several hundred thousand pixels and that each pixel is read sequentially, readout time is a major factor that limits the sampling rate of CCD systems. However, because of a larger number of recording sites (pixels), CCD detectors are better suited for mapping larger preparations at low magnification so that spatial resolution of 1 mm or less can be attained.

## Role Of Optical Mapping In The Understanding The Substrate For Cardiac Arrhythmias

In optical mapping, the time course of membrane potential is registered at every recording site. Therefore, it is possible to relate complex propagation patterns to voltage changes occurring at the cellular and subcellular level. Studies performed at the level of the single cell have shed important insights into the nature of intracellular propagation within single cardiomyocytes [9], as well as impulse propagation between cardiomyocytes [10], and have contributed greatly to our understanding of the role of cell-to-cell coupling, structural discontinuities, and tissue anisotropy in propagation of the electrical impulse [11]. More recently, optical mapping has also created new frontiers for investigating the physiology of very small but important structures such as the atrioventricular node [12].

Since reentrant cardiac arrhythmias may occur over various spatial scales, ranging from microreentry to rotors giving rise to spiral waves that encompass a whole atrium or ventricle, an effective mapping system should cover a relatively large area with closely spaced recording sites. Recent application of optical mapping techniques to the study of cardiac fibrillation has given us experimental proof of several theories of wave propagation in excitable media [13-17], such as the high-frequency reentrant sources that underlie fibrillation and generate spiraling waves that propagate throughout the ventricles in complex patterns [18]. Optical recording of the cardiac wavefront during reentry has demonstrated that conduction depends upon the curvature of the spiral wave, i.e., a greater degree of conduction slowing is noted in the presence of a more pronounced curvature. Cardiac excitation is enhanced when the curvature of the wavefront is less as compared to a highly convex wavefront [19,20] Using optical mapping, Laurita et al [21] have recently demonstrated how the spatial and temporal heterogeneity in action potential duration and repolarization lead to concordant and discordant alternans and ultimately reentrant arrhythmias.

## Recent Insights Into The Mechanisms Of Specific Arrhythmias

### AVNRT

**Figure 2:**
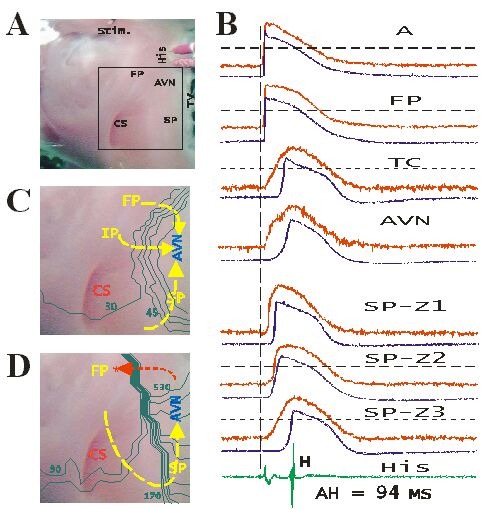
Using optical mapping to determine AV nodal electrophysiology. A: Method of optical mapping and microelectrode recording in isolated canine AV nodal preparation. The stimulation electrode (Stim.) and electrode recording His bundle electrogram (H) are located on the top portion of the picture. The square box next to the tricuspid valve (TV) represents the optical mapping area. B: optical (top) and intracellular action potentials recorded from different anatomic regions are superimposed. C: Activation map obtained during atrial pacing at cycle length of 700 ms shows three preferential AV nodal anterograde input pathways. D: Activation map obtained during a slow/fast type of AV nodal reentrant echo beat. Impulse was anterogradely conducted over the slow pathway and retrogradely conducted over the fast pathway. The star indicates the site of earliest retrograde atrial activation.

From our institution, Wu et al have recently defined the mechanisms underlying atrioventricular nodal conduction and the reentrant circuit of atrioventricular nodal reentrant tachycardia using optical mapping [12,22]. In contrast to long-standing concepts derived from traditional multisite mapping techniques-that propose the presence of discrete dual pathways within the atrioventricular node-optical mapping data suggest that atrial inputs into the AV node are from nondiscrete pathways, which can be arbitrarily divided into the fast (FP), intermediate (IP) and slow pathways (SP) (Figure 2C). In fact, the reentrant circuits of different types of AVNRT were observed directly. The reentrant circuit of the slow/fast type started counterclockwise with block in the FP, conduction delay in the SP connection to the compact AV node, then exit from the AV node to the FP, and rapid return to the SP through the atrial tissue located at the base of Koch’s triangle (Figure 2D). The reentrant circuit of the fast/slow type was clockwise. In the slow/slow type, anterograde conduction was over the IP and retrograde conduction was over the SP.

The new findings derived from these mapping data include the following: (1) the reentrant circuit for the slow/slow echo beat was different from that previously proposed. The term slow/slow type of AVNRT should be corrected to intermediate/slow type because multiple SPs were not observed to be involved. Thus, it is unlikely that the same SP could be used for anterograde and retrograde conduction. (2) Atrial tissue surrounding Koch’s triangle was clearly involved in all three types of AVNRT and suggested no upper common pathway. (3) Despite different reentrant circuits, the SP was always involved, explaining why ablation of the SP is effective in all types of AVNRT.

### Pulmonary Vein Electrophysiology

**Figure 3:**
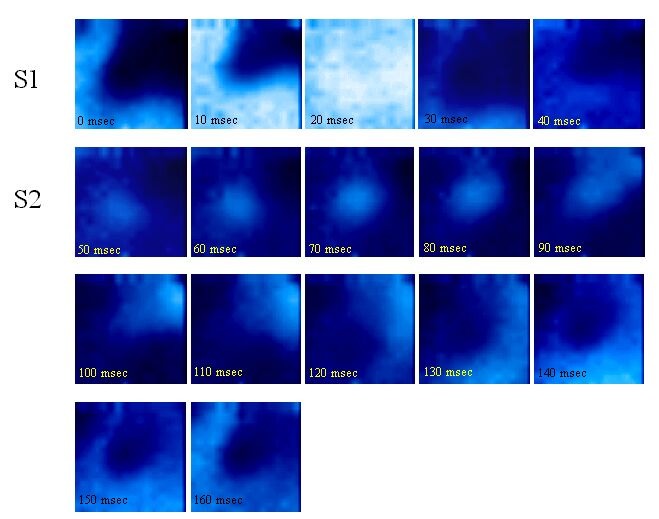
Reentry in the pulmonary vein. This figure demonstrates the progression of a reentrant wavefront around a zone of slow conduction at the venoatrial junction. Each image is a freeze frame at 10 msec increments. The first 3 frames (0-20 ms) show slow conduction at the venoatrial junction as the pacing stimulus (S1) progresses from the left side of the figure (atrium) to the right side (vein). Subsequent frames (30-160 ms) demonstrate a closely coupled extrastimulus (S2) that blocks at part of the venoatrial junction (lower right corner) but continues to propagate around the area of slow conduction, with a reentry cycle length of 140 msec. Modified from Arora et al. Arrhythmogenic substrate of the pulmonary veins assessed by high-resolution optical mapping. Circulation. 2003;107:1816-21

The pulmonary veins have recently been shown to harbor electrically active foci that can initiate and maintain atrial fibrillation [23] [24]. The electrophysiology of these focal “drivers” and “triggers” is not well understood however, in large part due to the inability to study this small region of the heart with traditional recording techniques. Very recently, we have utilized high-resolution optical mapping to study this hitherto obscure region, and have demonstrated regions of slow conduction as well as heterogeneity of repolarization within canine pulmonary veins [25]. In fact, the interplay of slow conduction and heterogeneous repolarization-best studied by a technique such as optical mapping, that allows simultaneous imaging of tissue activation of and repolarization-creates substrate for leading circle reentry within the pulmonary veins. Figure 3 shows an example of a reentrant beat, in response to an extrastimulus, with the entire cycle length shown as a propagation movie.

In this study, we also demonstrated focal activity within the pulmonary veins, the underlying mechanism of which appeared to be triggered activity. Both the epicardium as well as the endocardium of the pulmonary vein were studied; while reentry was seen only on the epicardial aspect of the vein, focal activity was seen only on the endocardium, close to the ostium of the vein.

## Direct Visualization Of VT Reentrant Circuit Using Optical Mapping

Reentry is the underlying mechanism for post-infarction ventricular tachycardia (VT) and the critical element of the reentrant circuit is the protected isthmus. Both anatomic and functional reentry have been proposed as potential models for such reentrant tachyarrhythmias. In anatomic reentry, the isthmus is fixed and located between two infarction scars or between an infarction scar and an anatomic obstacle such as the mitral valve annulus. In contrast, the isthmus in functional reentry is not fixed and is bounded by two functional lines of block located in the infarction border zone.

**Figure 4:**
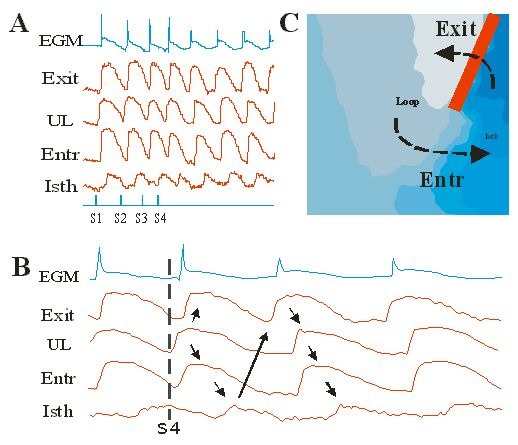
Sustained monomorphic VT induced by programmed stimulation. A. Selected optical action potentials from different sites during the initiation of VT are superimposed with extracellular bipolar electrograms (EGM). B. Selected portions of the same data shown in A are displayed at a faster sweep speed. C. The four components of reentrant circuit are superimposed with the activation map.

Using optical mapping in isolated canine left ventricular preparation, the reentrant circuit of monomorphic VT after acute myocardial infarction can be directly visualized (Figure 4). The reentrant circuit consisted of four components: 1) a isthmus with slow conduction, located within the border zone between the infarction area and the functional line of block; 2) an entrance site located at the beginning of the isthmus; 3) an exit site located at the other end of the isthmus; and 4) an outer loop consisting of a wide region of non-ischemic normal tissue, connecting the exit and entrant sites outside the border zone. These data suggest that the reentrant circuit is the combination of functional and anatomic reentry. This unique model might contribute to some of the VT seen in patients with myocardial infarction.

## Future Directions And Clinical Implications

As mentioned above, optical mapping was developed as a part of a quest to simultaneously map the activation as well as the recovery of electrically active tissues. The use of voltage-sensitive dyes, photodiodes, laser scanning and CCD cameras have made it possible to record electrical activity in tissue preparations as well as in a beating heart [26-28]. Several of the basic principles of clinical cardiac electrophysiology have been confirmed by optical mapping techniques, such as the demonstration that pacing at a faster cycle length shortens tissue repolarization, whereas delivery of a premature stimulus results in greater conduction slowing, resulting in a propensity towards genesis of cardiac re-entrant arrhythmias [21,29,30].

In addition to the insights that have been gained into the understanding of specific arrhythmias, the role of optical mapping has been recently extended by Garrigue and colleagues to study the effects of pacing in intact hearts. They studied the role of voltage output, interventricular delay, and pacing sites in ventricular arrhythmia occurrence during biventricular pacing (biventricular pacing is known to alleviate heart failure by restoring electrical, and therefore mechanical, synchrony between the ventricles); they utilized optical mapping in Langendorff-perfused guinea pig hearts to measure ventricular activation time and to examine conduction patterns during multisite pacing from 4 left ventricular and 3 right ventricular sites [27]. Myocardial ischemia was produced by gradually halving the perfusion output over 5 minutes. The optimal biventricular pacing sites were determined to be the RV apex and the base of the LV anterior wall; these sites were associated with the most homogeneous and rapid activation pattern (28 +/- 9 vs 41 +/- 12 ms with the other configurations, P < 0.01). There was no inducible arrhythmia. The role of high pacing output in the genesis of ventricular tachycardia was demonstrated in six hearts; ventricular tachycardia could be induced with pacing from the right and left free walls with 20 ms of interventricular delay, at six times the pacing threshold output. In four hearts, biventricular pacing (simultaneous right and left ventricular pacing) at high voltage output induced ventricular fibrillation with complex three-dimensional propagation patterns, independently of the pacing sites. The authors concluded that during biventricular pacing with ischemia, pacing at high voltage output with a long interventricular delay is likely to induce ventricular arrhythmias, particularly when left and right ventricular pacing results in a conduction pattern orthogonal to the ventricular myocardial fibers orientation. This study has indicated that it may be possible to record epicardial activation with optical mapping in determining the optimum site during left ventricular epicardial lead placement via thoracotomy. These experiments have also shown the feasibility of epicardial optical mapping in an intact heart and opened the way for further clinically relevant studies in. Although the mapping of endocardial activation in intact hearts is technically difficult at present, it is possible that with the advancement of echocardiographic techniques (such as intracardiac ultrasound) as well as further developments in optical mapping techniques, that intracardiac endocardial activation mapping may indeed become a reality in the not-too-distant future. Optical mapping is also expected to further our understanding the mechanisms of triggered and automatic activity, as well as the role of heterogeneous conduction and discordance of repolarization alternans in the genesis of reentry. A better understanding of the underlying mechanism of an arrhythmia would significantly help cardiologists tailor therapeutic strategies (whether ablative or pharmaceutical) to the individual patient.
